# TRENDS IN RISK FACTORS FOR DISABILITY AND REDUCED PHYSICAL FUNCTIONING AMONG US ADULTS DURING THE COVID-19 PANDEMIC

**DOI:** 10.1093/geroni/igac059.799

**Published:** 2022-12-20

**Authors:** Theresa Andrasfay, Eileen Crimmins

**Affiliations:** University of Southern California, Leonard Davis School of Gerontology, Los Angeles, California, United States; University of Southern California, Los Angeles, California, United States

## Abstract

The COVID-19 pandemic has caused widespread societal disruptions that may have impacted risk factors for disability and functional limitations. Current research suggests that physical inactivity, BMI, and smoking increased during the early phase of the pandemic, but less is known about whether these risk factors remained elevated as the pandemic progressed and mitigation measures were less disruptive. In this study, we assess trends in exercise frequency, BMI, and smoking among American adults aged 45 and over during the first year of the COVID-19 pandemic. We use data from the Understanding America Study (UAS) Coronavirus in America study, a nationally representative online survey that followed American adults from March 2020 to July 2021. We find that exercise frequency was highly seasonal throughout the pandemic, increasing during warmer months and decreasing during the colder winter months. Throughout this period, exercise frequency was highest among those with more education and those who were retired. Average BMI remained similar throughout this period, but less educated individuals and those working away from home and those who were unemployed experienced increases in BMI. Consistent with media reports suggesting increased interest in quitting smoking during the pandemic, we find decreases in smoking prevalence and frequency of smoking over this period. Decreases in smoking prevalence were concentrated among less educated individuals, who had higher prevalence of smoking to begin with. In sum, changes in risk factors for disability and reduced physical functioning during the pandemic were mixed and varied by education and employment status.

